# Processing of visual and non-visual naturalistic spatial information in the "parahippocampal place area"

**DOI:** 10.1038/s41597-022-01250-4

**Published:** 2022-04-01

**Authors:** Christian O. Häusler, Simon B. Eickhoff, Michael Hanke

**Affiliations:** 1 Psychoinformatics Lab, Institute of Neuroscience and Medicine, Brain & Behaviour (INM-7), Research Centre Jülich, Jülich, Germany; 2 Institute of Systems Neuroscience, Medical Faculty, Heinrich Heine University, Düsseldorf, Germany

**Keywords:** Perception, Language

## Abstract

The “parahippocampal place area” (PPA) in the human ventral visual stream exhibits increased hemodynamic activity correlated with the perception of landscape photos compared to faces or objects. Here, we investigate the perception of scene-related, spatial information embedded in two naturalistic stimuli. The same 14 participants were watching a Hollywood movie and listening to its audio-description as part of the open-data resource *studyforrest.org*. We model hemodynamic activity based on annotations of selected stimulus features, and compare results to a block-design visual localizer. On a group level, increased activation correlating with visual spatial information occurring in the movie is overlapping with a traditionally localized PPA. Activation correlating with semantic spatial information occurring in the audio-description is more restricted to the anterior PPA. On an individual level, we find significant bilateral activity in the PPA of nine individuals and unilateral activity in one individual. Results suggest that activation in the PPA generalizes to spatial information embedded in a movie and an auditory narrative, and may call for considering a functional subdivision of the PPA.

## Introduction

Studies in the field of neuropsychology and neuroimaging (e.g.,^1,2^) have shown that different parts of the brain are specialized for different perceptual and cognitive functions. The occipital cortex is considered to be primarily involved in the early stages of visual perception and giving rise to two distinct, but interacting, pathways that serve different functions: a) a dorsal stream (the “how pathway”) that leads into the parietal lobe and supports visual guidance of action, and b) a ventral stream (the “what pathway”) that leads into the temporal lobe and supports conscious perception and recognition^3–5^. A classic example of a higher-level visual area in the ventral pathway is the “parahippocampal place area” (PPA)^6,7^. The PPA is located in the posterior parahippocampal gyrus including adjacent regions of the fusiform gyrus and anterior lingual gyrus^8^. Increased hemodynamic activity is observed in the PPA when participants view pictures of landscapes, buildings or landmarks, compared to, e.g., pictures of faces or tools, during blood oxygenation level-dependent functional magnetic resonance imaging (BOLD fMRI) (see reviews^9,10^).

Increased hemodynamic activity in the PPA generalizes from pictures to mental imagery of landscapes^11^, haptic exploration of scenes constructed from LEGO blocks^12^, and scene-related sounds^13^. In a study conducted by O’Craven and Kanwisher^11^ participants viewed alternating blocks of pictures showing familiar places and famous faces during an initial experimental paradigm. In a subsequent paradigm, participants were instructed to “form a vivid mental image” of the previously viewed pictures. The PPA showed increased activation during imagination of places compared to faces but the imagination task showed a smaller activation level compared to the perceptual task. In a block design study conducted by Wolbers *et al*.^12^ the PPA of sighted as well as blind participants showed increased activation during a delayed match-to-sample task of haptically explored scenes constructed from LEGO bricks compared to abstract geometric objects.

To our knowledge only one study^14^ compared hemodynamic activity levels in the PPA that were correlated with different semantic categories occurring in *speech*. Aziz-Zadeh *et al*.^14^ used sentences that described famous or generic places, faces, or objects. Participants were instructed to press a button whenever the sentence described an inaccurate or improbable fact (e.g., “Marilyn Monroe has a large square jaw”). Activation in the left, but not right, PPA was significantly reduced when participants listened to place-related sentences compared to listening to face-related sentences. Moreover, this effect was only observed in sentences involving famous places.

Taken together, the literature suggests that the PPA does not exclusively respond to visually presented scene-related, spatial information. However, all reviewed studies share three common aspects: 1) they employed a small set of carefully chosen and conceptualized stimuli, 2) they exclusively used a block-design paradigm, and 3) they employed an explicit (perceptual) judgement task. Block-design studies that use conceptualized stimuli and a task have the advantage of controlling confounding variables (e.g., color, luminance, size, spatial frequencies, sentence length), maximizing detection power, and keeping participants paying attention to the stimuli. Nevertheless, small sets of conceptualized stimuli and block-design paradigms lack external and ecological validity^15,16^ because they poorly resemble how we, free of an explicit perceptual task, experience our rich, multidimensional and continuous environment that our brains are accommodated to^17^.

In this study, we investigate whether increased hemodynamic activity in the PPA that is usually detected by contrasting blocks of pictures is also present under more natural conditions. To answer this question, we operationalized the perception of both *visual and auditory* spatial information using two naturalistic stimuli (see reviews^17–19^). The current operationalization of visual spatial perception is based on an annotation of cuts and depicted locations in the audio-visual movie “Forrest Gump”^20^, while the operationalization of non-visual spatial perception is based on an annotation of speech occurring in the movie’s audio-description^21^. The movie stimulus shares the stimulation in the visual domain with classical localizer stimuli, while featuring real-life-like visual complexity and naturalistic auditory stimulation. The audio-description maintains the naturalistic nature of the movie stimulus, but limited to the auditory domain. We applied model-based, mass-univariate analyses to BOLD fMRI data from both naturalistic stimuli^22,23^, available from the open-data resource studyforrest.org. We compare current results to results of a previously performed model-based, mass-univariate analysis that was applied to data from a conventional functional localizer performed with the same set of participants^24^. Similarly to the functional localizer, we currently also capitalize on events that ought to evoke the cognitive processing of spatial information. Thus, we hypothesized that our whole-brain analyses would reveal increased hemodynamic activity in medial temporal regions that were functionally identified as the PPA by the analysis of the localizer data. We hypothesized further that a purely auditory stimulus could, in principle, localize the PPA as an example of a “visual area” in individual persons, and may offer an alternative paradigm to assess brain functions in visually impaired individuals.

## Results

We investigated if spatial information embedded in two naturalistic stimuli correlates with increased hemodynamic activity in the PPA. Based on an annotation of cuts in the movie and an annotation of speech spoken by the audio-description’s narrator, we selected events in both stimuli that should correlate with the perception of spatial information and contrasted them with events that should correlate with non-spatial perception to a lesser degree, or not at all. In order to test the robustness of our approach, we created multiple linear model (GLM) *t*-contrasts for the movie and audio-description. For each stimulus, we chose a primary contrast for result presentation based on a subjectively assessed balance of how well the averaged events within categories represent spatial and non-spatial information, and the number of events in the stimulus. On a group average level, we report results from whole-brain analyses of the movie’s and the audio-description’s primary contrasts and compare these results to a traditional visual localizer^24^. For each individual, we also compare the activation pattern from naturalistic auditory stimulation to the individual PPA localization with a block-design stimulus (see Figure S1 in the Supplementary Information for additional surface plots). An evaluation of the results’ robustness across all created contrasts on a group average level is provided in the Supplementary Information (see Figure S2). Unthresholded *Z*-maps of all contrasts on a group average level as well as individual results of the primary *t*-contrasts co-registered to the group-template (MNI152 space) can be found at neurovault.org/collections/KADGMGVZ.

### Group analyses

**Fig. 1:**
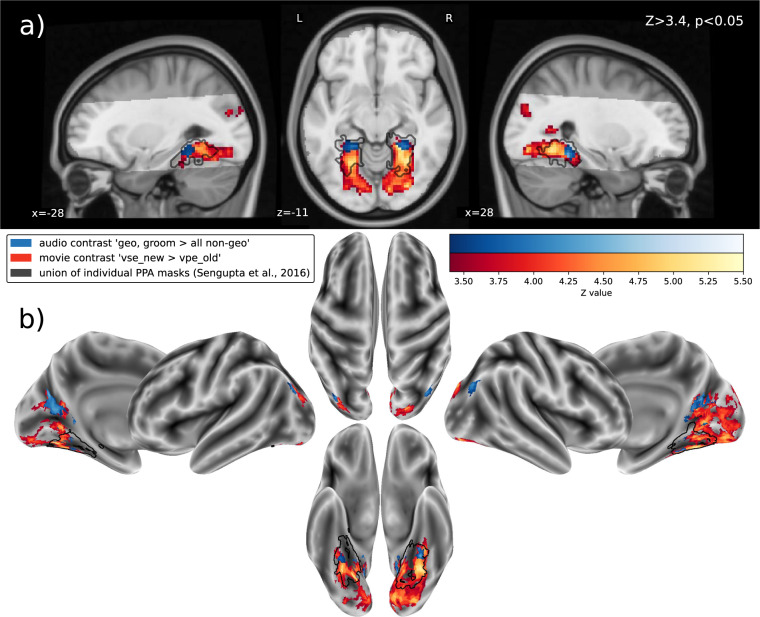
Mixed-effects group-level (N = 14) clusters (*Z* > 3.4; *p* < 0.05, cluster-corrected) of activity correlated with the processing of spatial information. The results of the audio-description’s primary *t*-contrast (blue) that compares geometry-related nouns spoken by the narrator to non-spatial nouns (geo, groom > all non-spatial categories) are overlaid on the movie’s primary *t*-contrast (red) that compares cuts to a setting depicted for the first time to cuts within a recurring setting (vse_new > vpe_old). (**a**) results as brain slices on top of the MNI152 T1-weighted head template, with the acquisition field-of-view for the audio-description study highlighted. For comparison depicted as a black outline, the union of the individual PPA localizations reported by Sengupta *et al*.^24^ that was spatially smoothed by applying a Gaussian kernel with full width at half maximum (FWHM) of 2.0 mm. (**b**) results projected onto the reconstructed surface of the MNI152 T1-weighted brain template. After projection, the union of individual PPA localizations was spatially smoothed by a Gaussian kernel with FWHM of 2.0 mm.

First, we analyzed data from the movie that offered ecologically more valid visual stimulation than a paradigm using blocks of pictures. The movie’s primary *t*-contrast that compared cuts to a setting that was not depicted before to cuts within a recurring setting (vse_new > vpe_old) yielded three significant clusters (see Table 1). Results are depicted as slices of brain volumes in Fig. 1a). The surface plots depicted in Fig. 1b) were created by reconstructing the cortical surface of the MNI152 template using FreeSurfer v7.1.1^25^, and projecting the *Z*-maps onto the surface using FreeSurfer’s’mri_vol2surf’ command. One cluster spans across the midline and comprises parts of the intracalcarine and cuneal cortex, the lingual gyrus and retrosplenial cortex, the occipital and temporal fusiform gyrus, and the parahippocampal cortex (reported from posterior to anterior) in both hemispheres. Two additional bilateral clusters are located in the superior lateral occipital cortex.

**Table 1:** Clusters (*Z*-threshold *Z* > 3.4; *p* < 0.05, cluster-corrected) of the primary *t*-contrast for the audio-visual movie comparing cuts to a setting depicted for the first time with cuts within a recurring setting (vse_new > vpe_old), sorted by size.

Voxels	${{\boldsymbol{p}}}_{{\boldsymbol{c}}{\boldsymbol{o}}{\boldsymbol{r}}{\boldsymbol{r}}{\boldsymbol{.}}}$	${{\boldsymbol{Z}}}_{{\boldsymbol{m}}{\boldsymbol{a}}{\boldsymbol{x}}}$	Max (MNI)			CoG (MNI)			Structure
			X	Y	Z	X	Y	Z	
3003	<0.00001	5.31	22.5	−45.5	−12	4.53	−63.3	−3.7	r. lingual g.; r. cuneal c., intracalcarine c., bilaterally occipital fusiform g., precuneus,temporal fusiform c., posterior parahippocampal c.
154	<0.00001	4.46	−35	−83	28	−32.8	−86.2	21.4	l. superior lateral occipital c.
121	<0.00001	4.65	25	−80.5	25.5	23.7	−83.8	25.4	r. superior lateral occipital cortex

The first brain structure given contains the voxel with the maximum *Z*-value, followed by brain structures from posterior to anterior, and partially covered areas (l.: left; r: right; c.: cortex; g.: gyrus; CoG: Center of Gravity).

Second, we analyzed data from the audio-description offering an exclusively auditory stimulation. The primary *t*-contrast for the audio-description (geo, groom > non-spatial noun categories) yielded six significant clusters (see Table 2, Fig. 1). Two bilateral clusters are located in the anterior part of the PPA group overlap reported by Sengupta *et al*.^24^. Specifically, these clusters are located at the borders of the posterior parahippocampal cortex, the occipital and temporal fusiform gyrus and lingual gyrus. Two additional bilateral clusters are apparent in the ventral precuneus extending into the retrosplenial cortex. Finally, two bilateral clusters are located in the superior lateral occipital cortex.

**Table 2:** Clusters (*Z*-threshold *Z* > 3.4; *p* < 0.05, cluster-corrected) of the primary *t*-contrast for the audio-description comparing geometry-related nouns to non-spatial nouns spoken by the audio-description’s narrator (geo, groom > all non-geo), sorted by size.

Voxels	${{\boldsymbol{p}}}_{{\boldsymbol{c}}{\boldsymbol{o}}{\boldsymbol{r}}{\boldsymbol{r}}{\boldsymbol{.}}}$	${{\boldsymbol{Z}}}_{{\boldsymbol{m}}{\boldsymbol{a}}{\boldsymbol{x}}}$	Max (MNI)			CoG (MNI)			Structure
			X	Y	Z	X	Y	Z	
188	<0.00001	4.48	−17.5	−65.5	25.5	−14.7	−59.1	15.2	l. precuneus
164	<0.00001	4.47	17.5	−58	23	15.6	−55.6	16	r. precuneus;
83	0.00013	4.48	27.5	−43	−17	27.2	−41.1	−14	r. occipito-temporal fusiform c.; posterior parahippocampal g.
73	0.00031	3.93	−22.5	−43	−12	−23.9	−43.6	−11.2	l. lingual g.; occipito-temporal fusiform g., posterior parahippocampal c.
63	0.00082	4.1	40	−75.5	30.5	40.9	−76.3	28.6	r. superior lateral occipital c.
37	0.0129	4.24	−37.5	−78	33	−38.4	−79.5	28.9	l. superior lateral occipital c.

The first brain structure given contains the voxel with the maximum *Z*-value, followed by brain structures from posterior to anterior, and partially covered areas (l.: left; r: right; c.: cortex; g.: gyrus; CoG: Center of Gravity).

### Individual analyses

**Fig. 2:**
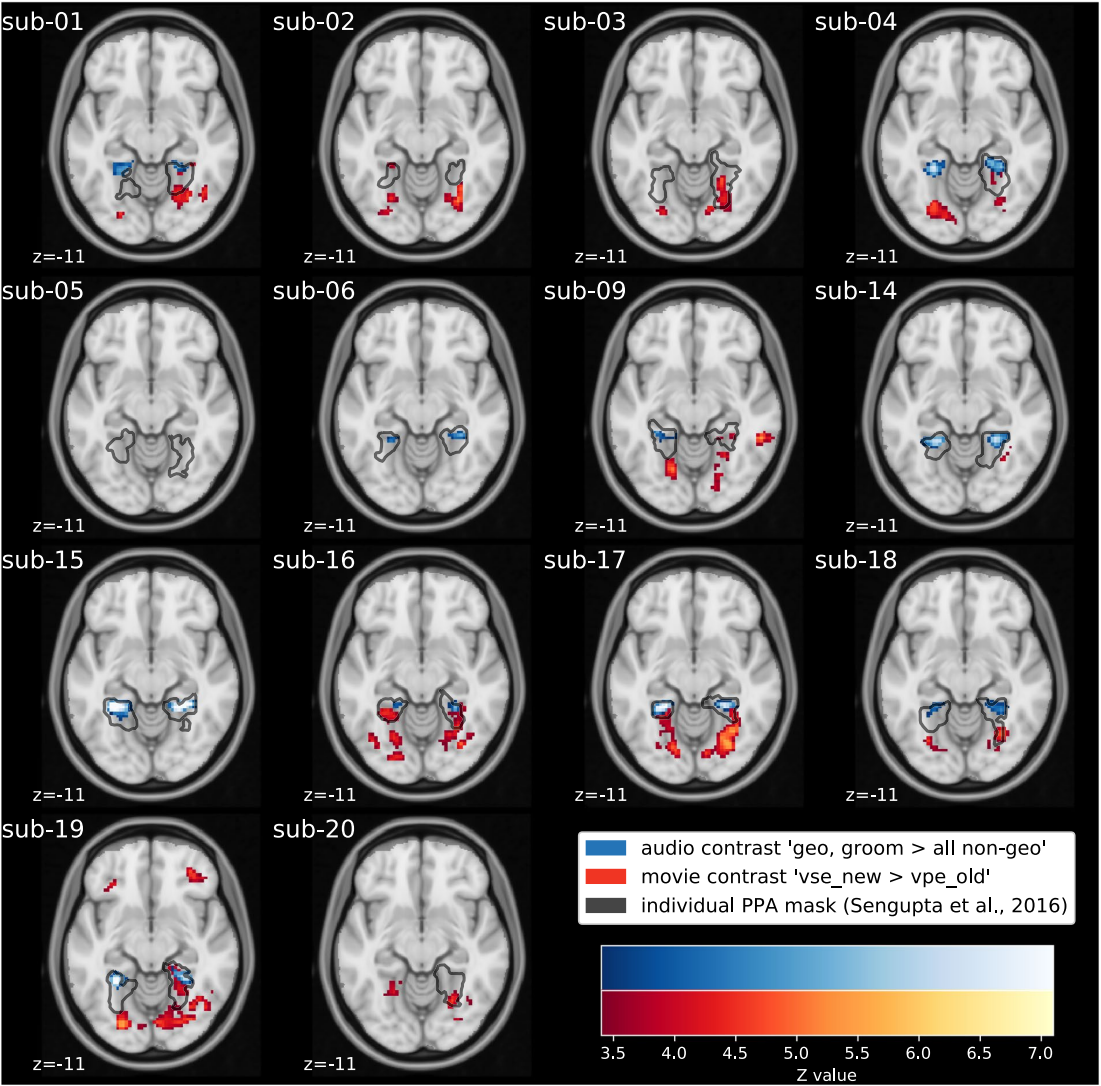
Fixed-effects individual-level GLM results (*Z* > 3.4; *p* < 0.05, cluster-corrected). Individual brains are aligned via non-linear transformation to a study-specific T2* group template that is co-registered to the MNI152 template with an affine transformation (12 degrees of freedom). The results of the audio-description’s primary *t*-contrast (blue) that compares geometry related nouns to non-geometry related nouns spoken by the narrator (geo, groom > all non-geo) are overlaid over the movie’s primary *t*-contrast (red) that compares cuts to a setting depicted for the first time with cuts within a recurring setting (vse_new > vpe_old). Black: outline of participant-specific PPA(s) reported by Sengupta *et al*.^24^. Light gray: The audio-description’s field of view^23^. To facilitate comparisons across participants, we chose the same horizontal slice (x = −11) for all participants as this slice depicts voxels of significant clusters in almost all participants. The figure does not show voxels of the left cluster of the movie stimulus in sub-09 and sub-18, and voxels of the right cluster of the movie stimulus in sub-15.

Third, we inspected results from both naturalistic paradigms on the level of individual participants, and compared them to the individual PPA localizations provided by Sengupta *et al*.^24^, who reported bilateral parahippocampal clusters in 12 of 14 participants and unilateral right clusters in two participants (see Table 3 in^24^). Unlike Sengupta *et al*.^24^, who determined PPA clusters using three candidate contrasts and a variable threshold, we used a single contrast for each naturalistic stimulus and a uniform threshold for all participants. Figure 2 depicts thresholded *Z*-maps of the primary movie and audio-description *t*-contrasts, in comparison to the results of a conventional block-design localizer (see Figure S1 in the Supplementary Information for surface plots; unthresholded *Z*-maps are provided at neurovault.org/collections/KADGMGVZ). Results of the primary movie contrast yielded bilateral clusters in five participants, a unilateral right cluster in six participants (of which one participant yielded a unilateral cluster in the visual localizer), and a unilateral left cluster in one participant. We find bilateral clusters for participant sub-20, whereas the block-design localizer yielded only one cluster in the right hemisphere. Results of the primary audio-description contrast yielded bilateral clusters in nine participants that are within or overlapping with the block-design localizer results. In participant sub-04, two bilateral clusters are apparent, whereas block-design localizer, and movie stimulus yielded only one cluster in the right hemisphere. For another participant (sub-09) the analysis yielded one cluster in the left-hemispheric PPA.

**Table 3:** Overview of event categories of the audio-visual movie and the audio-description.

Label	Description	All	1	2	3	4	5	6	7	8
*Movie stimulus*										
vse_new	change of the camera position to a setting not depicted before	96	11	14	17	4	17	9	21	3
vse_old	change of the camera position to a recurring setting	90	7	11	3	7	7	23	15	17
vlo_ch	change of the camera position to another locale within the same setting	89	10	31	2	23	0	4	18	1
vpe_new	change of the camera position within a locale not depicted before	386	31	38	72	90	89	33	24	9
vpe_old	change of the camera position within a recurring locale	208	25	61	0	13	1	32	29	47
vno_cut	frames within a continuous movie shot	148	30	13	0	21	15	27	9	17
fg_av_ger_lr	left-right luminance difference	180k	22k	22k	22k	24k	23k	22k	27k	16k
fg_av_ger_lrdiff	left-right volume difference	180k	22k	22k	22k	24k	23k	22k	27k	16k
fg_av_ger_ml	mean luminance	180k	22k	22k	22k	24k	23k	22k	27k	16k
fg_av_ger_pd	perceptual difference	180k	22k	22k	22k	24k	23k	22k	27k	16k
fg_av_ger_rms	root mean square volume	180k	22k	22k	22k	24k	23k	22k	27k	16k
fg_av_ger_ud	upper-lower luminance difference	180k	22k	22k	22k	24k	23k	22k	27k	16k
*Audio-description stimulus*										
body	trunk of the body; overlaid clothes	66	6	12	7	12	2	9	13	5
bpart	limbs and trousers	69	9	8	6	13	5	7	11	10
fahead	face or head (parts)	83	12	11	10	5	9	13	12	11
furn	moveable furniture (insides & outsides)	50	8	5	2	5	7	10	7	6
geo	immobile landmarks	125	16	17	11	32	0	15	18	16
groom	rooms & locales or geometry-defining elements	105	12	11	8	5	8	25	28	8
object	countable entities with firm boundaries	284	39	34	27	44	29	42	32	37
se_new	a setting occurring for the first time	86	11	15	12	4	15	10	16	3
se_old	a recurring setting	37	2	5	1	4	2	9	8	6
sex_f	female person(s), name	108	14	22	6	6	13	10	23	14
sex_m	male person(s), name	403	41	68	38	102	45	42	42	25
fg_ad_lrdiff	left-right volume difference	180k	22k	22k	21k	24k	23k	21k	27k	16k
fg_ad_rms	root mean square volume	180k	22k	22k	21k	24k	23k	21k	27k	16k

Event categories of the movie are based on an annotation of cuts and depicted locations. Event categories of the audio-description are based on an annotation of nouns spoken by the audio-description’s narrator (see Table 4). Some of the audio-description’s event categories listed here (sex_f; sex_m; fahead, object) were created by pooling some categories of the original annotation of nouns (female, females, fname; male, males, mname; face, head; object, objects). Respective event counts are given for the whole stimulus (All) and the segments that were used for the eight sessions of fMRI scanning. Event counts for frame-based features are reported in units of a thousand.

**Fig. 3:**
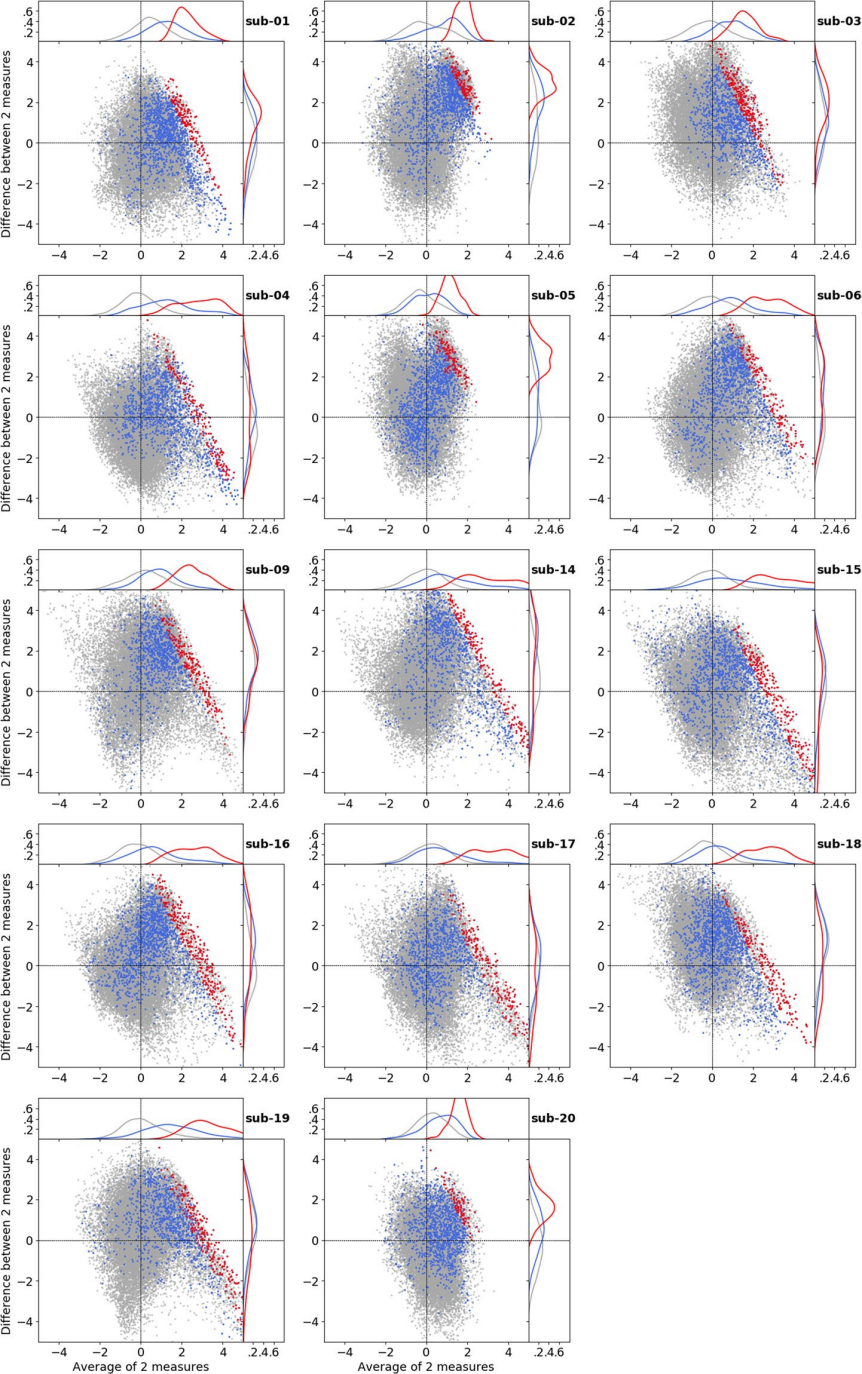
Bland-Altman-Plots for individual participants. The x-axes show the means of two spatially corresponding voxels in the unthresholded *Z*-map of the audio-description’s primary contrast and unthresholded *Z*-map of the visual localizer (KDE plot on the top). The y-axes show the difference of two voxels (localizer minus audio-description; KDE plot on the right). The overlays depict voxels spatially constrained to the temporal and occipital cortex (gray; based on probabilistic Jülich Histological Atlas^74,75^), PPA overlap of all participants (blue), and individual PPA(s) (red).

To illustrate the similarity of correlates of spatial processing in naturalistic (audio-description) and conventional stimulation, independent of a particular cluster-forming threshold, Bland-Altman plots for all participants are shown in Fig. 3. Each subplot visualizes the mean value and difference (localizer minus audio-description) of all voxels in temporal and occipital cortices. The marginal distributions of the mean scores indicate a general agreement of both contrast scores across voxels in the PPA localization overlap as a probabilistic indicator (blue), and the individual block-design localizer results (red). Notably, 11 participants exhibit a pattern of increased *Z*-scores for the naturalistic stimulus (lower right quadrant) that includes voxels labeled by the block-design localizer, but also additional voxels.

## Discussion

Several studies have reported increased hemodynamic activity in the PPA attributed to the processing of scene-related, spatial information, for example, when participants were watching static pictures of landscapes compared to pictures of faces or objects^6,7^. However, reports regarding the correlates of processing spatial information in verbal stimulation are less clear^14^. In line with previous studies, we investigated the hemodynamic response to spatial information using a model-based, mass-univariate approach. However, instead of using a conventional set of stimuli, assembled to specifically and predominantly evoke the processing of spatial information, we employed naturalistic stimuli, a movie and its audio-description, that were designed for entertainment.

We hypothesized that due to the complex nature of the stimuli, unrelated factors would be balanced across a large number of events, and make the bias of spatial information accessible to a conventional model-based statistical analysis of BOLD fMRI data. This model-driven approach required a detailed annotation of the occurrence of relevant stimulus features. The annotation of both stimuli revealed the respective number of incidentally occurring events to be similar to those of a conventional experimental paradigm used to localize functional regions of interest.

We modeled hemodynamic responses correlating with spatial information embedded in the naturalistic stimuli, capitalizing on conceptually similar, but perceptually different stimulation events. On a group-average level, results for the movie show significantly increased hemodynamic activity spatially overlapping with a conventionally localized PPA but also extending into earlier visual cortices. Likewise, results for the audio-description identify significant activation in the PPA but restricted to its anterior part. Bilateral clusters in 9 of 14 participants (of which sub-04 shows only a right-lateralized PPA in the block-design localizer results), and a unilateral significant cluster in one participant, indicate that the group average results are representative for the majority of individual participants. These findings suggest that increased activation in the PPA during the perception of static pictures generalizes to the perception of spatial information embedded in a movie or a purely auditory narrative. Current results may partially deviate from Sengupta *et al*.^24^, due to the uniform cluster forming threshold employed in this study versus their adaptive procedure (bilateral clusters in 12 of 14 participants and a unilateral right cluster in two participants (sub-04, sub-20).

The fact that clusters of responses to the auditory stimulus are spatially restricted to the anterior part of clusters from both visual paradigms raises the question if the revealed correlation patterns can be attributed to different features inherent in the visual stimuli compared to a purely auditory stimulus. Due to the nature of the datasets investigated here, such an attribution can only be preliminary, because the auditory stimulation dataset also differs in key acquisition properties (field-strength, resolution) from the comparison datasets, representing a confound of undetermined impact. However, previous studies in the field of visual perception provide evidence that the PPA can be divided into functionally subregions that might process different stimulus features. The posterior PPA (pPPA) is functionally more responsive than the anterior PPA (aPPA) to low-level features of scenes or (abstract) objects^26–29^. In contrast, the aPPA responds more to high-level features of scenes (e.g., real-word size^30^; a scene’s abstract category or context^31,32^) and objects (e.g., spatial contextual associations^10,33^) than the pPPA. Moreover, pPPA and aPPA show differences in connectivity profiles. The pPPA exhibits more coactivation with the occipital visual cortex than the aPPA^26,34^. Activity in the aPPA, on the other hand, is found to be correlated with components of the default mode network, including caudal inferior parietal lobe, retrosplenial complex, medial prefrontal cortex, and lateral surface of the anterior temporal lobe^26,34^. Baldassano *et al*.^26^ propose that the PPA creates a complete scene representation, based on different aspects of a visual scene processed in subregions of the PPA. Similarly, our results suggest that the pPPA might be more concerned with visual spatial features that are intrinsic to pictures or movie shots of landscapes. The aPPA, in contrast, might be more concerned with spatial information that cannot only be inferred from visual stimuli but also from auditory stimuli, such as speech. For example, scene properties extrapolated from a context description or label, such as “football stadium”, “military hospital”, or objects and their spatial relationship, such as the descriptions “bus stop” and “roadside”, or the sound of a car passing by.

Based on the report by Aziz-Zadeh *et al*.^14^, we hypothesized that semantic spatial information embedded in the audio-description would correlate with increased hemodynamic activity in the PPA. Methods and results presented here differ from this previous study in key aspects. Aziz-Zadeh *et al*.^14^ modeled events from onset to offset of sentences, describing unknown and famous places and faces, and compared activity levels that were averaged across voxels of regions of interest (PPA and fusiform face area, FFA^35^) defined by a localizer experiment. Their results showed decreased activity in only the left PPA compared to activity in the FFA for sentences describing famous places in contrast to famous faces. Here, we modeled events from onset to offset of single words and performed a voxelwise whole-brain analysis. Group results of the audio-description’s primary contrast yielded significantly increased hemodynamic activity spatially restricted to the anterior part of the PPA group overlap. Contrary to Aziz-Zadeh *et al*.^14^, our results suggest that auditory spatial information compared to non-spatial information correlates with bilaterally increased activation in the anterior part of the PPA.

It is common for conventional localizer paradigms to employ a task to keep participants attentive to the stimuli. Nevertheless, one early block-design study^6^ compared results from a paradigm that employed a perceptual judgment task of static pictures to the same paradigm but without that task. Hemodynamic activity was less but still significantly increased when participants had no task to keep them alert and attentive to the stimuli. The naturalistic stimulation paradigm employed here is similar in the sense that participants had no behavioral or cognitive task (e.g., forming a mental image of the stimuli^11^) but just had to “enjoy the presentation”. Nevertheless, the naturalistic paradigm differs from Epstein and Kanwisher^6^, because the relevant stimulus features were embedded in a continuous stream of complex auditory (and visual) information which makes it unlikely that participants speculated on the purpose of the investigation, or performed undesired and unknown evaluation or categorization of isolated stimuli. Our results therefore indicate that verbally communicated spatial information is processed, in the anterior PPA, automatically and without specifically guided attention.

Our approach to movie stimulus annotation and event selection differs from previous reports in the literature. In an earlier study, Bartels and Zeki^36^ manually annotated the content of movie frames (color, faces, language, and human bodies) and found that the functional specialization of brain areas is preserved during movie watching. In contrast, we aimed to exploit a cinematographic confound in the structure of movies, where film directors tend to establish the spatial layout of locations in earlier shots and later focus more on detailed depictions of people and objects^37–39^. The group results of the movie stimulus’ primary contrast yielded a large cluster that spans the group overlap of individual PPAs from anterior to posterior. The cluster extends into more posterior, earlier visual areas could be an indication that the temporal averaging across events suffered from insufficient controlling for confounding visual features. Future studies that aim to use a movie to localize visual areas in individual participants should extensively annotate the content of frames (e.g., using the open-source solution “Pliers”^40^ for feature extraction from a visual naturalistic stimulus).

In the visual domain, pictures of landscapes and not pictures of landmarks or buildings are considered to be the “optimal” stimulus type^8^. In the audio-description’s primary contrast, we did not include the categories that contain switches from one setting to another (se_new and se_old) which one might assume to contain the auditory equivalent to pictures of landscapes. The reason was that the categories se_new and se_old were heterogeneous: they rarely contained holistic (but also vague) descriptions of landscapes (e.g., “[Forrest is running through the] jungle”) but mostly landmarks or buildings, and also non-spatial hints (e.g., “[Jenny as a] teenager“). Humans can identify the gist of a rich visual scene within the duration of a single fixation^41^. Hence, further studies might investigate if vague verbal descriptions of landscapes lead to a different hemodynamic activity level than descriptions of more concrete parts of a scene (e.g., “[a] beacon”, “[a] farmhouse”).

Apart from the PPA, results show significantly increased activity in the ventral precuneus and posterior cingulate region (referred to as “retrosplenial complex”, RSC) of the medial parietal cortex, and in the superior lateral occipital cortex (referred to as “occipital place area”, OPA) for both naturalistic stimuli. Like the PPA, the RSC and OPA have repeatedly shown increased hemodynamic activity in studies investigating visual spatial perception and navigation^42–45^. Thus, our model-driven approach to operationalize spatial perception based on stimulus annotations reveals increased hemodynamic responses in a network that is implicated in visual spatial perception and cognition. Similarly to the parahippocampal cortex^10^, the medial parietal cortex exhibits a posterior-anterior gradient from being more involved in perceptual processes to being more involved in memory related processes^42,46–48^. Future, complementary studies using specifically designed paradigms could investigate where in the posterior-anterior axis of the parahippocampal and medial parietal cortex auditory semantic information is correlated with increased hemodynamic activity: we hypothesize that the auditory perception of spatial information (compared to non-spatial information) is correlating with clusters in the middle of possibly overlapping clusters correlating with visual perception (peak activity more posterior) and scene construction from memory (peak activity more anterior).

In summary, natural stimuli like movies^17,19,49^ or narratives^18,50–53^ can be used as a continuous, complex, immersive, task-free paradigm that more closely resembles our natural dynamic environment than traditional experimental paradigms. We took advantage of three fMRI acquisitions and two stimulus annotations that are part of the open-data resource studyforrest.org to operationalize the perception of spatial information embedded in an audio-visual movie and an auditory narrative, and compare current results to a previous report of a conventional, block-design localizer. The current study offers evidence that a model-driven GLM analysis based on annotations can be applied to a naturalistic paradigm to localize concise functional areas and networks correlating with specific perceptual processes – an analysis approach that can be facilitated by the neuroscout.org platform^54^. More specifically, our results demonstrate that increased activation in the PPA during the perception of static pictures generalizes to the perception of spatial information embedded in a movie and an exclusively auditory stimulus. Our results provide further evidence that the PPA can be divided into functional subregions that coactivate during the perception of visual scenes. Finally, the presented evidence on the in-principle suitability of a naturally engaging, purely auditory paradigm for localizing the PPA may offer a path to the development of diagnostic procedures more suitable for individuals with visual impairments or conditions like nystagmus.

## Methods

We used components of the publicly available studyforrest.org dataset that has been repeatedly used by other research groups in independent studies (e.g.,^55–59^). The same participants were a) listening to the audio-description^23^ of the movie “Forrest Gump”, b) watching the audio-visual movie^22^, and c) participating in a dedicated six-category block-design visual localizer^24^. An exhaustive description of the participants, stimulus creation, procedure, stimulation setup, and fMRI acquisition can be found in the corresponding publications. Following is a summary of the most important aspects.

### Participants

In the audio-description study^23^, 20 German native speakers (all right-handed, age 21–38 years, mean age 26.6 years, 12 male) listened to the German audio-description^60^ of the movie “Forrest Gump”^61^. In the movie study^22^, 15 participants (21–39 years, mean age 29.4, six female), a subgroup of the prior audio-description study, watched the audio-visual movie with dubbed German audio track^62^. In the block-design localizer study^24^, the same 15 participants took part in a six-category block-design visual localizer. All participants reported to have normal hearing, normal or corrected-to-normal vision, and no known history of neurological disorders. In all studies, participants received monetary compensation and gave written informed consent for their participation and for public sharing of obtained data in anonymized form. The studies had prior approval by the Ethics Committee of Otto-von-Guericke University of Magdeburg, Germany.

### Stimuli and procedure

The German DVD release^62^ of the movie “Forrest Gump”^61^ and its temporally aligned audio-description^60^ served as naturalistic stimuli, with an approximate duration of two hours, split into eight consecutive segments of ≈15 minutes. The audio-description adds another male narrator to the voice-over narration of the main character Forrest Gump. This additional narration describes essential aspects of the visual scenery when there is no off-screen voice, dialog, or other relevant auditory content. For all sessions with naturalistic stimuli, participants were instructed to inhibit physical movements except for eye-movements, and otherwise to simply “enjoy the presentation”. For details on stimulus creation and presentation see Hanke *et al*.^22,23^.

Stimuli for the block-design localizer study were 24 unique grayscale images of faces, bodies, objects, houses, outdoor scenes and scrambled images, matched in luminance and size, that were previously used in other studies (e.g.,^63^). Participants performed a one-back image matching task for four block-design runs, with two 16 s blocks per stimulus category in each run. For details on stimulus creation and presentation see Sengupta *et al*.^24^.

### Stimulation setup

In the audio-description study, visual instructions were presented on a rear-projection screen inside the scanner bore. During the functional scans, the projector presented a medium gray screen with the primary purpose to illuminate a participant’s visual field in order to prevent premature fatigue. In the movie and block-design localizer study, visual instructions and stimuli were presented on a rear-projection screen at a viewing distance of 63 cm, with a movie frame projection size of approximately 21.3° × 9.3°. In the block-design localizer study, stimulus images were displayed at a size of approximately 10° × 10° of visual angle. Auditory stimulation was implemented using custom in-ear (audio-description), or over-the-ear headphones (movie), which reduced the scanner noise by at least 20–30 dB.

### fMRI data acquisition

Gradient-echo fMRI data for the audio-description study were acquired using a 7 Tesla Siemens MAGNETOM magnetic resonance scanner equipped with a 32 channel brain receive coil at 2 s repetition time (TR) with 36 axial slices (thickness 1.4 mm, 1.4 × 1.4 mm in-plane resolution, 224 mm field-of-view, anterior-to-posterior phase encoding direction) and a 10% inter-slice gap, recorded in ascending order. Slices were oriented to include the ventral portions of frontal and occipital cortex while minimizing intersection with the eyeballs. The field of view was centered on the approximate location of Heschl’s gyrus. EPI images were online-corrected for motion and geometric distortions.

In the movie and block-design localizer study, a 3 Tesla Philips Achieva dStream MRI scanner with a 32 channel head coil acquired gradient-echo fMRI data at 2 s repetition time with 35 axial slices (thickness 3.0 mm, 10% inter-slice gap) with 80 × 80 voxels (3.0 × 3.0 mm of in-plane resolution, 240 mm field-of-view) and an anterior-to-posterior phase encoding direction, recorded in ascending order. A total of 3599 volumes were recorded for each participant in each of the naturalistic stimulus paradigms (audio-description and movie).

### Preprocessing

The current analyses were carried out on the same preprocessed fMRI data (s. github.com/psychoinformatics-de/studyforrest-data-aligned) that were used for the technical validation analysis presented in Hanke *et al*.^22^. Of those 15 participants in the studyforrest dataset that took part in all three experiments, data of one participant were dropped due to invalid distortion correction during scanning of the audio-description stimulus. Data were corrected for motion, aligned with and re-sliced onto a participant-specific BOLD template image^24^ (uniform spatial resolution of 2.5 × 2.5 × 2.5 mm for both audio-description and movie data). Preprocessing was performed by FEAT v6.00 (FMRI Expert Analysis Tool^64^) as shipped with FSL v5.0.9 (FMRIB’s Software Library^65^) on a computer-cluster running NeuroDebian^66^.

For the present analysis, the following additional preprocessing was performed. High-pass temporal filtering was applied to every stimulus segment using a Gaussian-weighted least-squares straight line with a cutoff period of 150 s (sigma = 75.0 s) to remove low-frequency confounds. The brain was extracted from surrounding tissues using BET^67^. Data were spatially smoothed applying a Gaussian kernel with full width at half maximum (FWHM) of 4.0 mm. A grand-mean intensity normalization of the entire 4D dataset was performed by a single multiplicative factor. Correction for local autocorrelation in the time series (prewhitening) was applied using FILM (FMRIB’s Improved Linear Model^64^) to improve estimation efficiency.

### Event selection

In contrast to stimuli designed to trigger a perceptual process of interest, while controlling for confounding variables (e.g., color and luminance), naturalistic stimuli have a fixed but initially unknown temporal structure of stimulus features of interest, as well as an equally unknown confound structure. In order to evaluate the suitability of the stimulus for the targeted analyses, and to inform the required hemodynamic models, we annotated the temporal structure of a range of stimulus features.

For the analysis of the movie stimulus, we took advantage of a previously published annotation of 869 movie cuts and the depicted location after each cut^20^. Contrary to manually annotating stimulus features of movie frames (for example, as performed by Bartels and Zeki^36^ for color, faces, language, and human bodies), we categorized movie cuts that - in general - realign the viewer within the movie environment by switching to another perspective within the same setting, or to a position in an entirely different setting. More specifically, we sought to exploit a cinematographic bias as at a setting’s first occurrence in a movie, shots tend to broadly establish the setting and the spatial layout within the setting. On revisiting an already established setting, the shot sizes tend to decrease and more often depict people talking to each other or objects that are relevant to the evolved plot^37–39^.

Based on this cinematographic bias, we assigned each cut to one of five categories (see Table 3): 1) a cut switching to a setting that was depicted for the first time (vse_new), 2) a cut switching to a setting that was already depicted earlier in the movie (vse_old), 3) a cut switching to another locale within a setting (vlo_ch; e.g., a cut from the first to the second floor in Forrest’s house), 4) a cut to another camera position within a setting or locale that was depicted for the first time (vpe_new), and 5) a cut to another camera position within a setting or locale that was already depicted before (vpe_old). Note that this categorization is not necessarily evident from the visual content of the first movie frame after a cut. As a control condition with events of no particular processing of spatial information (no_cut) we pseudo-randomly selected movie frames from continuous movie shots that lasted longer than 20 s, and had a minimum temporal distance of at least 10 s to any movie cut and to any other no_cut event.

For the analysis of the audio-description stimulus, we extended a publicly available annotation of its speech content^21^ by classifying concrete and countable nouns that the narrator uses to describe the movie’s absent visual content. An initial annotation was performed by one individual, and minor corrections were applied after comparing with a second categorization done by the author. A complete overview of all 18 noun categories, their inclusion criteria, and examples can be seen in Table 4. Some categories reflect the verbal counterpart of the stimulus categories that were used in the visual localizer experiment (e.g., body, face, head, object, setting_new, and setting_rec). Other categories were created to semantically cluster remaining nouns into categories that had no counterpart in the visual localizer experiment (e.g., bodypart, female, fname, furniture, geo, groom, male, and persons). The categories setting_new and setting_rec comprise not just words that describe a setting as a whole (e.g., “[in] Greenbow”, “[in a] disco”, “[the platoon wades through a] rice field”). They also comprise words that could count as a member of another category in case the narrator uses these words to indicate a switch from one setting to another (e.g., “[a] physician”). These nouns were flagged with both categories during the initial annotation procedure. In context of the current analyses these word are considered to belong exclusively to the higher-level category of changing a setting (setting_new or setting_rec). Some noun categories that were semantically similar and offered only a small amount of counts were pooled resulting in 11 final event categories (see Table 3). These event categories were then used to model hemodynamic responses.

**Table 4:** Categories and criteria to categorize the nouns spoken by the audio-description’s narrator.

Category	Criteria	Examples
body	trunk of the body; possibly clothed	back, hip, shoulder; jacket, dress, shirt
bodypart	limbs	arm, finger, leg, toe
face	face or parts of it	face, ear, nose, mouth
female	female person	nurse, mother, woman
females	female persons	women
fname	female name	Jenny
furniture	movable furniture (insides & outsides)	bench, bed, table, chair
geo	immobile landmarks	building, tree, street, alley, meadow, cornfield
groom	rooms & locales, or geometry-defining elements	living room; wall, door, window, floor
head	non-face parts of the head; worn headgear	head, hair, ear, neck; helmet
male	male person	man, father, soldier
males	male persons	boys, opponents
mname	male name	Bubba, Kennedy
object	countable entity with firm boundaries	telephone, car
objects	countable entities	wheels, plants
persons	concrete persons of unknown sex	hippies, patients
setting_new	a setting occurring for the first time	on a “bridge”, on an “alley”, on “campus”
setting_rec	a recurring setting	at the “bus stop”

Examples are given in English. Some of these initial 18 noun categories were pooled resulting in 11 event categories that served as basis to build the regressors of the GLM (see Table 3).

Lastly, we algorithmically annotated the temporal structure of low-level perceptual features to create nuisance regressors. For the movie stimulus, we computed the mean luminance (arbitrary units, average pixel brightness) of a movie frame (40 ms), the difference in mean luminance of each frame’s left and right half, the difference in mean luminance of the lower and upper half. As an indicator of visual change, we computed perceptual fingerprints for each movie frame using the pHash library^68^ and recorded the cumulative bitwise difference to the previous frame. Finally for both audio tracks, we computed the left-right difference in volume and root mean square volume averaged across the length of every movie frame (40 ms).

### Hemodynamic modeling

The events of the movie cut related categories and no_cut events were modeled as box car events of 200 ms duration, speech events from onset to offset of each word, and frame-based confounds from onset to offset of the corresponding movie frame. For all regressors, the stimulus models were convolved with a double-gamma hemodynamic response function (HRF), as implemented in FSL.

**Fig. 4:**
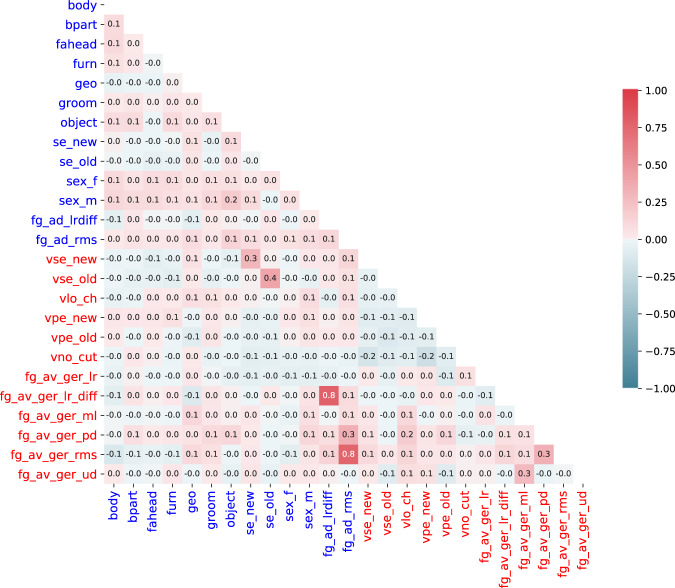
Pearson correlation coefficients of model response time series used as regressors in the GLM analysis of the audio-description (blue; see Table 3 for a description) and audio-visual movie (red; see Table 3). Values are rounded to the nearest tenth. The correlation between the two stimuli’s root mean square volume and between their left-right difference in volume yielded the highest correlation values (fg_ad_rms and fg_av_ger_rms, r = 0.7635; fg_ad_lrdiff and fg_av_ger_lrdiff, r = 0.7749).

Given the unknown confound structure of the naturalistic stimuli, we inspected the correlation of regressors of each stimulus and also across both stimuli (see Fig. 4). The computed Pearson correlation coefficients show only minor correlations of feature regressors for the same stimulus. Maximum correlations are found for low-level auditory confounds across stimuli, which is to be expected, as the audio-description contains the audio track of the movie plus the additional narrator (see Fig. 4; correlation between root mean square volume of both audio tracks, r = 0.76; correlation between left-right volume difference of both audio tracks, r = 0.77). The observed correlation pattern did not indicate problematic confounds of nuisance variables and regressors of interest for any of the planned analyses.

The design matrix for the first-level time-series GLM analysis of the movie comprised regressors for the 12 event categories of the movie listed in Table 3. Similarly, the design matrix for the analysis of the audio-description comprised regressors for the 13 event categories of the audio-description, also listed in Table 3. In order to implement cross-modal control contrasts, the design matrix for the movie stimulus also contained the regressors based on nouns used by the audio-description’s narrator to indicate a switch to another setting (categories se_new and se_old). Likewise, the design matrix for the audio-description included the five movie cut related regressors (vse_new, vse_old, vlo_ch, vp_new, vpe_old). For both stimuli, a null regressor was used for any event category in a segment for which no event of a category was present (e.g., no event of vpe_old in segment 3; see Table 3). Temporal derivatives of all regressors were added to the design matrix to compensate for regional differences^69^. Lastly, six participant-specific and run-specific motion estimates (translation and rotation) were also included. The design matrices were temporally filtered with the same high-pass filter (cutoff period of 150 s) as the BOLD time series.

### Statistical analysis

We performed standard two-level GLM analyses to aggregate results across all BOLD acquisition runs for every participant and each stimulus separately. Subsequently, we conducted third-level analyses to average contrast estimates over participants for each stimulus, respectively.

Given the rich naturalistic stimuli and the availability of various stimulus feature annotations, there are several candidates for implementing contrasts to identify hemodynamic activity congruent with the hypothesized processing of spatial information in the PPA. However, while the rationale for any contrast composition must be sound, the selection of a single implementation remains arbitrary to some degree. Consequently, we computed multiple contrasts that are listed in Table 5 for the movie and audio-description, respectively. For each stimulus, we picked a primary contrast for result presentation (marked with an asterisk in the table) based on a subjectively assessed balance of how well the averaged events within categories represent spatial and non-spatial information, and the number of events in the stimulus. An evaluation of the robustness of these results with respect to similar but different contrasts is provided in the Supplementary Information.

**Table 5:** Computed contrasts for the analysis of the movie and the audio description, and their respective purpose.

Nr.	Contrast	Purpose
*Movie stimulus*		
1*	vse_new > vpe_old	spatial processing
2	vse_new, vpe_new > vse_old, vpe_old	spatial processing
3	vse_new > vse_old	spatial processing
4	vse_new > vse_old, vpe_old	spatial processing
5	vse_new, vpe_new > vpe_old	spatial processing
6	vno_cut > vse_new	control
7	vno_cut > vse_old	control
8	vno_cut > vse_new, vse_old	control
9	vno_cut > vpe_new, vpe_old	control
10	se_new > se_old	control (absent narrator)
*Audio-description stimulus*		
1*	geo, groom > non-spatial	spatial processing
2	geo, groom, se_new > non-spatial	spatial processing
3	groom, se_new, se_old > non-spatial	spatial processing
4	geo > non-spatial	spatial processing
5	groom > non-spatial	spatial processing
6	se_new > non-spatial	spatial processing
7	se_new, se_old > non-spatial	spatial processing
8	se_new > se_old	spatial processing
9	vse_new > vpe_old	control (absent visual cuts)
10	vse_new, vpe_new > vse_old, vpe_old	control (absent visual cuts)
11	vse_new > vse_old	control (absent visual cuts)
12	vse_new > vse_old, vpe_old	control (absent visual cuts)
13	vse_new, vpe_new > vpe_old	control (absent visual cuts)

The primary contrasts are marked with an asterisk. non-spatial refers to the event categories body, bodypart, fahead, object, sex_f, sex_m. An explanation of all event categories can be found in Table 3.

In the primary contrast of the movie stimulus, we contrasted cuts to a setting that was not depicted before (vse_new) to cuts merely switching the camera position within a setting that was already established earlier in the movie (vpe_old). We chose the contrast vse_new > vpe_old as the primary contrast, because it is contrasting the most different categories in regard to the averaged movie frames’ content: due to a cinematographic bias, the category vse_new tends to comprise mostly shots that were designed to orient the viewer in the movie’s broader environment using wider shots sizes (ranging from “establishing shots” to “wide shots”). In contrast, the category vpe_old comprises mostly shots depicting details of a scene using narrower shot sizes (ranging from “medium shots” to “close ups”). audio-description stimulus, we contrasted mentions of buildings and landmarks (geo), and rooms and geometry-defining objects (groom) with nouns referring to a person’s head or face (fahead), and other non-geometry related categories (body, bodypart, fahead, object, sex_f, sex_m). The category “furniture” (furn) was not used as a regressor of interest in any contrast because these nouns could be perceived as either a geometry-defining scene element or as an isolated object. The categories of nouns indicating a switch to a setting that occurred for the first time (se_new) or a setting that recurred during the plot (se_old) were not included in the primary contrast because they comprise a) nouns that could be a member of another category (e.g., “[a] physician”), and b) nouns that only vaguely identify a scene (e.g., “[in] Greenbow”, “[in a] disco”, ”[black and white] film recordings”) compared to perceptually richer descriptions.

Finally, we created several control contrasts for both stimuli (see Table 5). For the movie stimulus, four contrasts were created contrasting the no-cut regressor (vno_cut) to cut-related regressors. One contrast was created by contrasting nouns spoken by the (missing) narrator comparing nouns that indicate a switch to another setting (se_new > se_old), and that were moderately correlated with movie cuts indicating a switch to another setting (see Fig. 4). For the audio-description stimulus, we created five control contrasts to test if activation in the PPA was correlated with moments of cuts, despite the absence of visual stimulation.

First-level GLM analyses were performed in the image-space of a participant-specific BOLD T2* template using a previously determined linear transformation^24^. For higher-level analyses image data were aligned with a study-specific T2* group template likewise using a previously computed non-linear transformation^23^. This group template was co-registered to the MNI 152 template with an affine transformation (12 degrees of freedom).

The second-level analyses, which averaged contrast estimates over the eight stimulus segments, were carried out using a fixed-effects model by forcing the random effects variance to zero in FLAME (FMRIB’s Local Analysis of Mixed Effects^70,71^). (Gaussianised T/F) statistic images were thresholded using clusters determined by *Z*>3.4 and a cluster-corrected significance threshold of *p* = 0.05^72^.

The third level analysis which averaged contrast estimates over participants was carried out using FLAME stage 1 with automatic outlier detection^70,71,73^. Here again, Z (Gaussianised T/F) statistic images were thresholded using clusters determined by *Z* > 3.4 and a corrected cluster significance threshold of *p* = 0.05^72^. Brain regions associated with observed clusters were determined with the Jülich Histological Atlas^74,75^ and the Harvard-Oxford Cortical Atlas^76^ provided by FSL. Regions of interest masks for individual PPAs and a PPA group mask of individual PPA overlaps were created from data provided by Sengupta *et al*.^24^.

## Supplementary information

Supplementary Information

## Data Availability

All fMRI data, annotations, and results are available as Datalad^77^ datasets, published to or linked from the *G-Node GIN* repository (10.12751/g-node.7is9s6^78^). Raw data of the audio-description, movie and visual localizer were originally published on the *OpenfMRI* portal (https://legacy.openfmri.org/dataset/ds000113^79^, https://legacy.openfmri.org/dataset/ds000113d^80^). Results from the localization of higher visual areas are available as Datalad datasets at *GitHub* (https://github.com/psychoinformatics-de/studyforrest-data-visualrois). The realigned participant-specific timeseries that were used in the current analyses were derived from the raw data releases and are available as Datalad datasets at *GitHub* (https://github.com/psychoinformatics-de/studyforrest-data-aligned). The annotation of cuts is available at *F1000Research* (10.5256/f1000research.9536.d134823^81^), the annotation of speech is available at *OSF* (10.17605/OSF.IO/F5J3E^82^). Participant-specific reconstructed cortical surface are available as Datalad dataset at *GitHub* (https://github.com/psychoinformatics-de/studyforrest-data-freesurfer), and are also part of the original data release on *OpenfMRI* (https://legacy.openfmri.org/dataset/ds000113^79^). The same data are available in a modified and merged form on OpenNeuro at https://openneuro.org/datasets/ds000113. Unthresholded *Z*-maps of all contrasts can be found at neurovault.org/collections/KADGMGVZ.

## Code availability

Scripts to generate the results as Datalad^77^ datasets are available in a *G-Node GIN* repository (10.12751/g-node.7is9s6^78^).
