# Supplementary material for: Processing of visual and non-visual naturalistic spatial information in the "parahippocampal place area"
Source: Sci Data. 2022 Apr 1;9:147. doi: 10.1038/s41597-022-01250-4 (PMC8975992; doi:10.1038/s41597-022-01250-4)
Supplement: Supplementary file 1 — Supplementary Information [file 41597_2022_1250_MOESM1_ESM.pdf]

Processing of visual and non-visual naturalistic spatial information in the "parahippocampal place area"

Supplementary Information

Christian O. Häusler[1,2*], Simon B. Eickhoff[1,2], Michael Hanke[1,2]

February 14, 2022

1. Psychoinformatics Lab, Institute of Neuroscience and Medicine, Brain & Behaviour (INM-7), Research Centre Jülich, Jülich, Germany
2. Institute of Systems Neuroscience, Medical Faculty, Heinrich Heine University, Düsseldorf, Germany
*corresponding author: Christian Olaf Häusler (der.haeusler@gmx.net)

Contents

1 Surface plots of individual results **2**

2 Robustness of main findings across alternative contrasts **4**

3 Incidental results **6**

4 Control contrasts **6**

List of Figures

S1 Participant-specific surface plots of primary contrasts 3
S2 Group results across contrasts of movie and audio-description 5

1 Surface plots of individual results

The results of the fixed-effects second-level analysis of the movie's primary contrast (`vse_new > vpe_old`) and audio-description's primary contrast (`geo, groom` > non-spatial noun categories) mapped onto subject-specific cortical surfaces can be seen in Figure S1. FreeSurfer v5.3.0[1] was used to reconstruct surfaces from T1-weighted images and an additional high-resolution T2-weighted image (s. `github.com/psychoinformatics-de/studyforrest-data-freesurfer`). Thresholded Z-maps (Z>3.4; p<.05, cluster-corrected) were projected onto the cortical surfaces using FreeSurfer's[1] 'mri_vol2surf' command (unthresholded Z-maps are provided at `neurovault.org/collections/KADGMGVZ`). After projection, individual PPA ROIs were spatially smoothed by applying a Gaussian kernel with full width at half maximum (FWHM) of 2.0 mm.

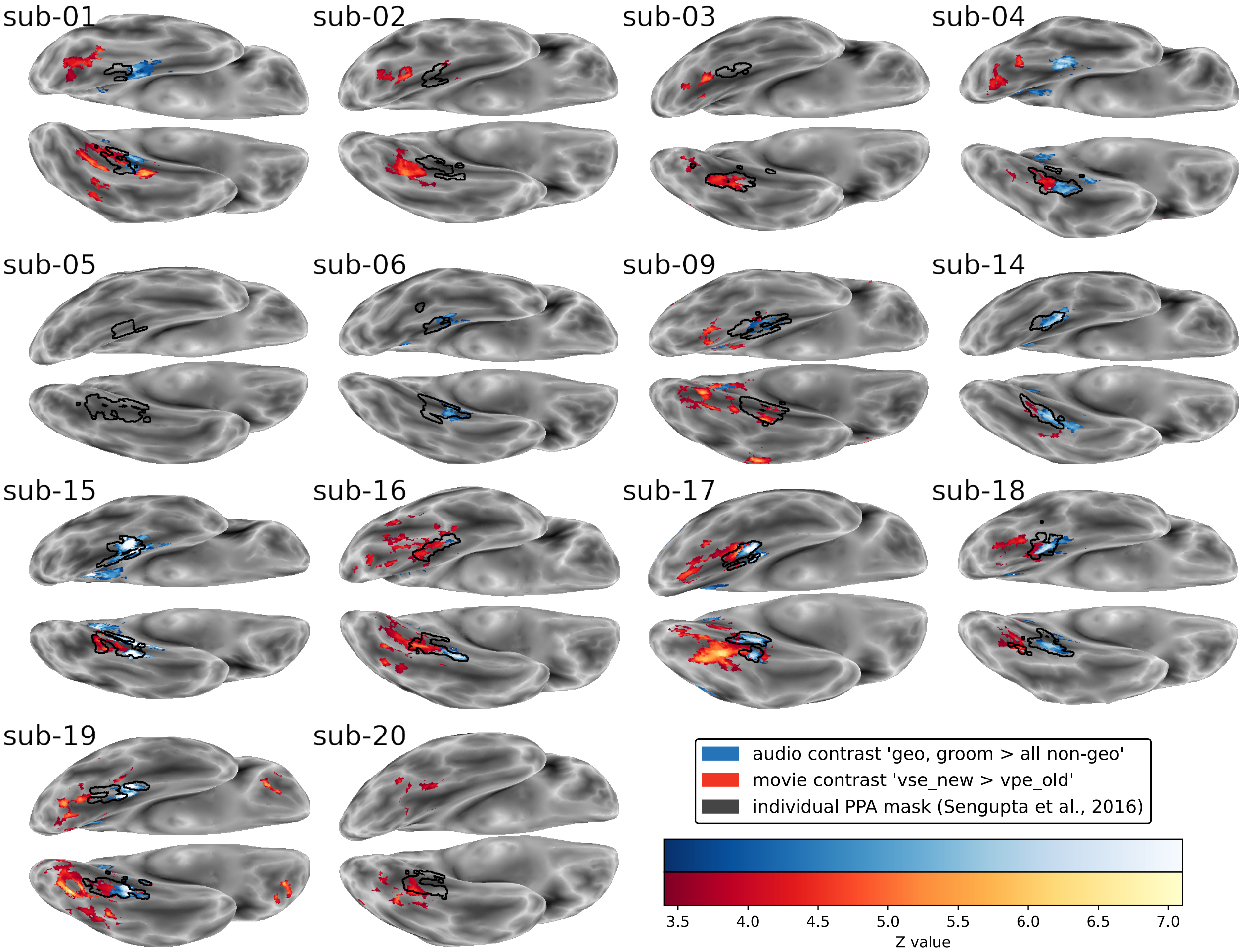

Figure S1: Fixed-effects individual-level GLM results (Z>3.4; p<.05 cluster-corrected) projected onto reconstructed subject-specific brain surfaces. The results of the audio-description's primary t-contrast (blue) that compares geometry related nouns to non- geometry related nouns spoken by the narrator (`geo, groom > all non-geo`) are overlaid over the movie's primary t-contrast (red) that compares cuts to a setting depicted for the first time with cuts within a recurring setting (`vse_new > vpe_old`). Black: outline of participant-specific PPA ROIs reported by Sengupta et al.[2] that was spatially smoothed by applying a Gaussian kernel with full width at half maximum (FWHM) of 2.0 mm.

2 Robustness of main findings across alternative contrasts

In order to test the robustness of our approach, we created overall five t-contrasts for the movie stimulus, and overall eight t-contrast for the audio-description (see Table 5). Contrasts differ in contrasted categories based on "how well" averaged events within categories were considered to represent spatial and non-spatial information, and the number of events in the stimulus. Unthresholded Z-maps of all contrasts on a group level co-registered to the group-template (MNI152 space) can be found at `neurovault.org/collections/KADGMGVZ`.

Reliably across all five movie contrasts, we find bilaterally significant clusters that overlap with the PPA group overlap (see Figure S2). Several contrasts of the movie also yielded bilaterally significant clusters in the ventral precuneal cortex and posterior cingulate gyrus (i.e. retrosplenial complex; contrasts 1, 4, 5), and lateral occipital cortex (contrasts 1, 2, 4, 5).

In results from the analysis of the audio-description, all contrasts except contrast 7 and 8 yielded significant bilateral clusters in anterior regions of the group PPA overlap (see Figure S2). Contrasts of the audio-description also yielded bilaterally significant clusters in the ventral precuneus (all contrasts except contrasts 8) and lateral occipital cortex (bilateral in contrasts 1 to 4; unilateral in contrasts 6 and 7). Results across contrasts of both naturalistic stimuli indicate that our findings regarding the PPA but also RSC and LOC are robust and do not depend on the design of one specific contrasts. Nevertheless, results are sensitive to the contrasted categories and the amount of available data. For example, contrast 7 and 8 (`se_new`, `se_old` > non-spatial categories, and `se_new` > non-spatial categories) that used the most heterogeneous categories (nouns indicating switches to other settings) and a low amount of events yielded neither a significant cluster in the right-hemispheric nor left-hemispheric PPA. Hence, investigators that use model-driven analyses have to consider how many events a naturalistic stimulus may provide and how homogeneous the events to be averaged might be.

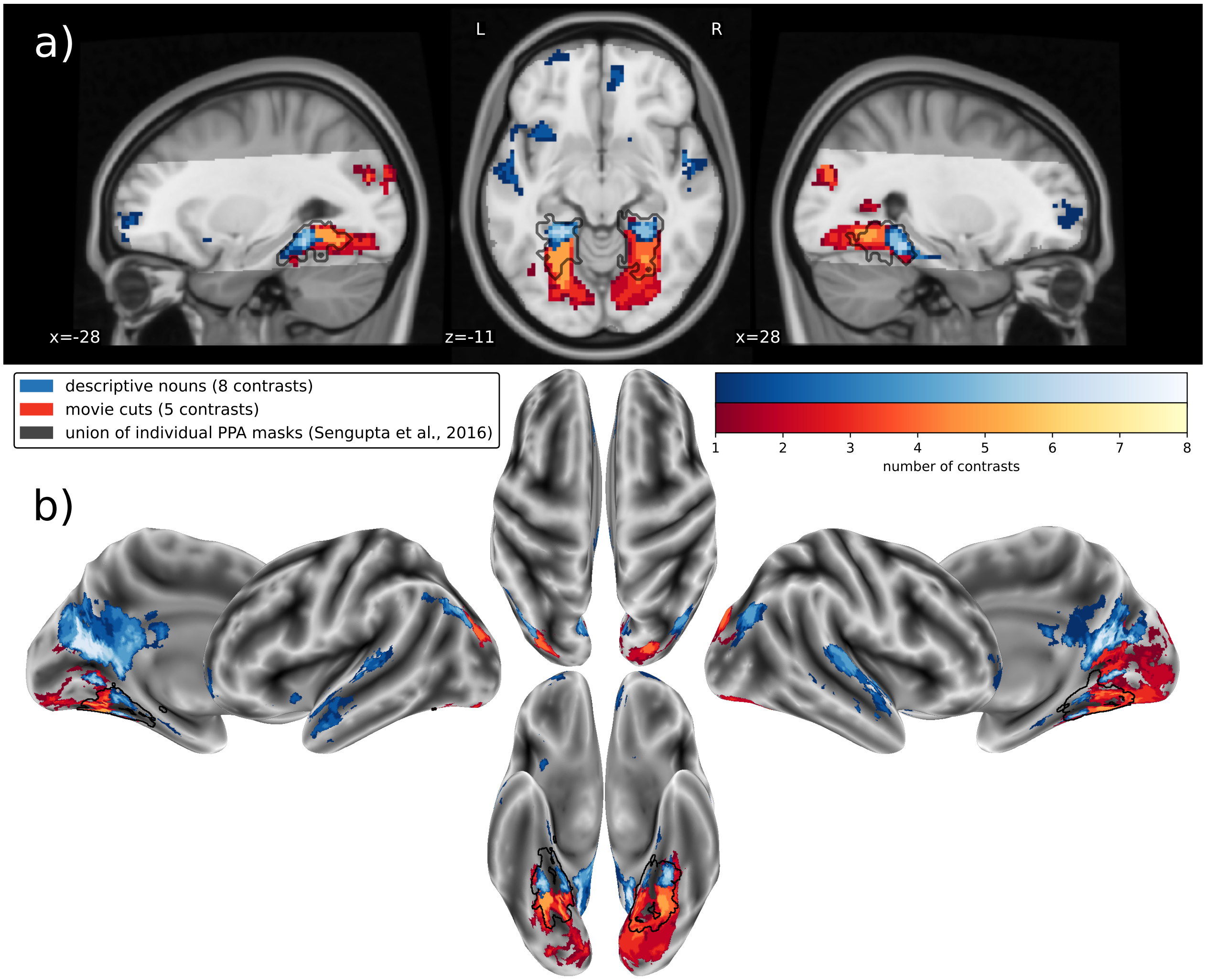

Figure S2: Overlap of significant clusters (Z>3.4; p<.05, cluster corrected) across all contrasts for both naturalistic stimuli. The audio-description's contrasts 1-8 (blue) are overlaid over the audio-visual movie's contrasts 1-5 (red; see Table 5) . a) results as brain slices on top of the MNI152 T1-weighted head template, with the acquisition field-of-view for the audio-description study highlighted. For comparison depicted as a black outline, the union of the individual PPA localizations reported by Sengupta et al.[2] that was spatially smoothed by applying a Gaussian kernel with full width at half maximum (FWHM) of 2.0 mm. b) results projected onto the reconstructed surface of the MNI152 T1-weighted brain template. After projection, the union of individual PPA localizations was spatially smoothed by a Gaussian kernel with FWHM of 2.0 mm

3 Incidental results

Several contrasts yielded significant clusters in superior temporal cortices (bilateral in contrasts 3, 6, 7; unilateral right in contrast 2). Further, contrasts that compare nouns indicating a switch to another setting (categories `se_new` or `se_old`) with nouns from non-spatial categories yielded significant clusters in primary and secondary auditory cortices (bilateral: contrasts 3, 6, 7; unilateral right: contrast 2). This suggests the presence of a low-level auditory processing confound that was not completely captured by the employed nuisance regressors. The bias of the GLM contrasts towards low-level auditory perceptual processes might be attributed to changes in the soundscape or the start of a new song that accompany the narrator when he is indicating a switch to another setting.

Lastly, contrasts of the audio-description stimulus yielded clusters in regions that were classified as statistically significant in two or less contrasts. In detail, contrast 2 yielded clusters in the anterior cingulate gyrus (bilateral), right frontal pole, right frontal medial cortex, and right putamen. Contrast 3 shows clusters in the anterior cingulate gyrus (bilateral), left frontal pole, and left insular. Contrast 6 shows one additional cluster in the right medial frontal cortex. Contrast 7 shows clusters in the left frontal pole and left frontal operculum, left insular. Here again, significantly increased activation beyond areas known to be involved in processing of spatial information could be attributed to stimulus features of the naturalistic stimulus that were not sufficiently controlled leading to a bias in the contrasts.

4 Control contrasts

For each naturalistic stimulus, we created several control contrasts. To test for the specificity of the main findings within the movie fMRI data, we created four contrasts that compared hemodynamic activity during frames within movie shots (`no_cut` category) with movie cut related categories (see Table 5). None of these four contrasts yielded a significant cluster, suggesting that the observed differential hemodynamic response in the PPA for events defined by particular movie cuts is not observed for other arbitrarily selected time points in the movie.

The correlation of regressors across stimuli (Figure 4) showed a moderate correlation among cuts to a setting depicted for the first time (movie `vse_new`) and nouns indicating a switch to a setting occurring for the first time (audio-description `se_new`), and cuts to a recurringly depicted setting (movie `vse_old`) and nouns indicating a switch a recurring setting (audio-description `se_old`). Hence, we computed an indentical contrast (`se_new` > `se_old`), comparing the audio-description's voice-over narrator nouns for the movie data and the audio-description. While for the movie data the contrast yielded a significant cluster in the right PPA, not in its anterior portion, and bilateral clusters in the superior lateral occipital cortices (the right hemispheric cluster extending into the superior parietal cortex), no significant differences where found for the audio-description (unthresholded contrast maps are available on NeuroVault). These results suggest that in the absence of the actual visual stimulation, the narrator's description of scene properties correlated with setting changes did not co-occur with increased hemodynamic activity in areas associated with the processing of spatial information.

Finally for the analysis of the audio-description, we computed control contrasts based on events of the annotated movie cuts (see Table 5) in order to evaluate the influence of stimulus features correlated with these visual events that remain present in the audio-description, such as changes in the soundscape. Two of five contrasts yielded significant clusters. Contrast 9 (`vse_new > pe_old`) revealed one significant cluster in the left inferior prefrontal cortex (pars triangularis) and right ventral precuneus/posterior cingulate gyrus. Contrast 12 (`vse_new` > `vse_old`, `vpe_old`) revealed one cluster in the right posterior hippocampus. The absence of observable hemodynamic response differences in the PPA suggests that without the visual information in the movie stimulus, no, or substantially less, processing of stimulus content with respect to spatial information co-occurred with the timing of the movie cuts.

References

[1] Dale, A. M., Fischl, B. & Sereno, M. I. Cortical surface-based analysis: I. Segmentation and surface reconstruction. *NeuroImage* **9**, 179–194 (1999).

[2] Sengupta, A. *et al.* A studyforrest extension, retinotopic mapping and localization of higher visual areas. *Sci. Data* **3** (2016).
